# Pregnancy Outcomes of Women With Polycystic Ovary Syndrome for the First *In Vitro* Fertilization Treatment: A Retrospective Cohort Study With 7678 Patients

**DOI:** 10.3389/fendo.2020.575337

**Published:** 2020-09-25

**Authors:** Su Liu, Meilan Mo, Shan Xiao, Longfei Li, Xiuyu Hu, Ling Hong, Linlin Wang, Ruochun Lian, Chunyu Huang, Yong Zeng, Lianghui Diao

**Affiliations:** Shenzhen Key Laboratory of Reproductive Immunology for Peri-implantation, Shenzhen Zhongshan Institute for Reproduction and Genetics, Shenzhen Zhongshan Urology Hospital, Shenzhen, China

**Keywords:** polycystic ovary syndrome, *in vitro* fertilization, pregnancy complications, pregnancy outcomes, body mass index

## Abstract

**Background:**

The risk of adverse pregnancy outcomes is increased by having a polycystic ovary syndrome (PCOS) diagnosis. However, the confounders in previous studies preclude firm conclusions, and further studies are warranted.

**Objectives:**

To investigate whether PCOS affects pregnancy outcomes and complications in infertile women undergoing their first *in vitro* fertilization (IVF) treatment, taking into account important confounders.

**Methods:**

We performed a retrospective cohort study of 7,678 infertile women, including 666 women with PCOS and 7,012 controls undergoing their first IVF treatment at a private fertility center from January 2010 to December 2017. Our main outcome was the impact of PCOS on adverse pregnancy outcomes (miscarriage, preterm delivery, pregnancy-induced hypertension) and pregnancy outcomes (live birth rate, clinical pregnancy rate, implantation rate). PCOS effects were summarized by adjusted odds ratios (aORs) with 95% confidence intervals (CIs) after controlling for maternal characteristics.

**Results:**

After adjusting for differences in maternal age, BMI, infertility duration, total dose of gonadotropin, serum E_2_ and endometrial thickness on the day of hCG trigger, number of fertilized occytes, number of embryos transferred, embryo type (cleavage-stage embryo or blastocyst) and quality, women with PCOS had an increased risk of developing unfavorable pregnancy complications, including miscarriage (aOR 1.629, 95% CI 1.240–2.141), very preterm delivery (< 32 weeks) (aOR 2.072, 95% CI 1.133–3.791). For pregnancy outcomes, PCOS was associated with higher clinical pregnancy rate (aOR 1.248, 95% CI 1.038–1.501) and implantation rate (aOR 1.238, 95% CI 1.030–1.489) after adjusting for the above-mentioned confounders.

**Conclusions:**

Women with PCOS are at increased risk of adverse pregnancy outcomes after adjusting for differences in maternal characteristics. These women may need more frequent medical consultants and management during pregnancy and parturition.

## Introduction

Polycystic ovary syndrome (PCOS) is one of the most common endocrine disorders that affects 5%–15% in women of reproductive age, depending on the population studied and the diagnostic criteria used (1). The most common features of PCOS are oligo- or anovulation, hyperandrogenism, and polycystic ovaries (2). Other endocrinal disorders such as insulin resistance and obesity play essential roles in the pathogenesis of PCOS (3, 4). These endocrinal diseases are also known risk factors to induce metabolic syndrome, pregnancy loss and late pregnancy complications, which indicates that PCOS is a chronic disease with manifestations across the lifespan (5).

Previous studies have reported an increased risk of pregnancy complications, such as gestational diabetes mellitus (GDM), pregnancy-induced hypertension (PIH), and preterm delivery in the PCOS population (6–8). However, the pathophysiological explanation is debated, as in these studies, several characteristics of the PCOS population, including maternal age, body mass index (BMI) and the use of assisted reproductive technologies may independently contribute to adverse pregnancy outcomes and thus confound the study conclusions. To our knowledge, there have been very few studies on pregnancy outcomes in women with PCOS, accounting for all the relevant confounders. Consequently it is uncertain considering the extent to which the risk of adverse pregnancy outcomes in women with PCOS is attributed to the underlying disorder. Therefore, to drive a more precise evaluation of the risks of pregnancy complications in women with PCOS, we conducted this retrospective study to investigate the prevalence of adverse pregnancy outcomes in women with PCOS by comparison with control group after adjusting for other relevant confounders.

## Materials and Methods

### Participants

We identified 9,074 patients from Shenzhen Zhongshan Urology Hospital (SZUH) who had their first IVF treatment between January 2010 and December 2017. From the original cohort, we excluded patients who had virus infection (including HBV, HCV, HIV, and syphilis) (n=160). Patients aged > 38 years (n=561) and those treated with gonadotropin-releasing hormone (GnRH)-antagonist controlled ovarian hyperstimulation (COH) protocols (n=196) were also excluded. We also excluded the cycles missing embryo information and clinical pregnancy data, patients suffering from a chromosomal abnormality, intrauterine death, a medical abortion, stillbirth, or ectopic pregnancy (n=479). PCOS was diagnosed if patients meet two or three of the following criteria: (1) oligo- or amenorrhoea; (2) biochemical or clinical hyperandrogenism; (3) multi-follicular appearance of ovaries on transvaginal ultrasound scan, according to the Rotterdam criteria. Oligomenorrhea was defined as less than 8 cycles/year or menstrual interval > 35 days, whereas amenorrhoea was defined as absence of menses in the last 6 or more months. Clinical hyperandrogenism was defined by the Ferriman-Galwey score more than 6 (9), whereas biochemical hyperandrogenism was defined by a total T more than or equal to 0.481 ng/ml. The polycystic ovarian morphology (PCOm) was defined as ≥ 12 follicles in either ovary, measuring 2–9 mm in diameter and/or increased ovarian volume of each ovary > 10 ml on ultrasound scan (10). Finally, the study population consisted of 666 women diagnosed with PCOS and 7,012 controls (**Supplemental Figure 1**). Pregnancies from patients with congenital adrenal hyperplasia, Cushing’s syndrome and androgen-secreting tumors, nonclassic adrenal hyperplasia, thyroid dysfunction, hyperprolactinemia, type 2 diabetes mellitus or cardiovascular disease were excluded. All controls had regular menstrual cycles and normal androgen levels, none had polycystic ovaries on ultrasound. No subjects had received hormonal treatment or insulin-lowering agents in the previous quarter. The Institutional Review Board of Reproductive Research Ethics Committees of Shenzhen Zhongshan Urology Hospital approved this study (Approval number: SZZSECHU-20180030). Due to the retrospective nature of the study, the requirement of informed consent was waived.

### Laboratory Tests

All blood samples were collected in the morning after an overnight (8–10 h) fast and preferably on Days 2–5 of the spontaneous menstrual cycle in regularly menstruating women or during withdrawal bleeding in amenorrheic women. Blood samples were aliquoted for plasma insulin, TSH, total T4, LH, FSH, total T, plasma glucose, blood counts, and liver and kidney functions. Serum were separated at room temperature and aliquoted as per the requirements. The aliquots were stored at −70°C until the assay. All hormonal assays were carried out by chemiluminescence under Cobas e601 (Roche Diagnostics, Germany) using commercial kits. Plasma glucose and other biochemical parameters were assayed on Cobas c501 autoanalyzer (Roche Diagnostics, Germany). For all measurements, the inter- and intra-assay coefficient of variations were within the limits permitted by the manufacturers. To investigate insulin sensitivity, the quantitative insulin sensitivity check index (QUICKI) was calculated. QUICKI=1/[log(I_0_)+log(G_0_)], where I_0_ is the fasting insulin, and G_0_ is the fasting glucose. QUICKI is a validated surrogate marker for insulin resistance and has good agreement with gold standard hyperinsulinaemic euglycaemic clamp (11).

### IVF and Embryo Transfer Protocols

All patients’ information, including their baseline characteristics, cycle characteristics, and pregnancy outcomes were extracted from the electronic medical record. All included patients underwent IVF cycles receiving a routine luteal phase down-regulation protocol with GnRH agonist protocol. The gonadotropin starting dose and the GnRH analog were selected based on the physician’s discretion. Monitoring of ovarian response was assessed by serum E_2_ levels and transvaginal ultrasound scan. Final oocyte maturation was induced by injection of human chorionic gonadotropin (hCG) when at least two follicles reached 17–18 mm. Transvaginal ultrasound guided oocyte retrieval was carried out 36 h following hCG injection. Traditional IVF or intracytoplasmic sperm injection (ICSI) was performed as indicated. Embryo quality was evaluated by two experienced embryologists based on the number of blastomeres and the percentage of fragmentation. Ultrasound-guided fresh embryo transfer was performed on day 3 or day 5 after fertilization.

### Measurement of Outcomes

Pregnancy outcomes including implantation rate, clinical pregnancy rate and live birth rate were collected and measured. Adverse pregnancy outcomes such as miscarriage, multiple pregnancies, preterm delivery, GDM, PIH were reported and the incidence of each complication was measured. The implantation rate was calculated by the number of intrauterine gestational sacs per transferred embryo. Clinical pregnancy was defined by the sonographic presence of a gestational sac at 7–8 weeks of gestation. Live birth rate was calculated as cycles with delivered live babies divided by the number of total embryo transferred cycles. Miscarriage included early miscarriage (pregnancy loss before 12 weeks of gestation) and late miscarriage (pregnancy loss within 12–28 weeks of gestation). Preterm delivery was defined as delivery at < 37 completed gestational weeks and stratified into very preterm delivery (< 32 weeks) and moderately preterm birth (32–36 weeks). GDM was performed at 24 weeks of gestation and defined as plasma glucose levels > 10 mmol/L after a 2-h oral glucose tolerance test with 75 g of glucose. PIH was described as hypertension with a systolic blood pressure over 140 mm Hg or diastolic blood pressure over 90 mm Hg, with or without proteinuria, developing after 20 weeks of gestation.

### Statistical Analysis

Descriptive statistical analysis was performed on main maternal and cycle characteristics. Continuous data with normal distribution were presented as the mean ± standard deviation (SD) and analyzed by independent *t*-test. The continuous variables that did not show a normal distribution were presented as median and interquartile range, and were analyzed by Mann-Whitney *U*-test. Categorical data were presented by the number of cases and corresponding percentage, and were analyzed by Pearson’s χ^2^ test or Fisher’s exact test.

To assess the relationships between PCOS and different pregnancy outcomes, logistic regression analysis, adjusted for potential confounding covariates, were performed. The selection of variables was based on identifying all measured clinical variables of known or suspected prognostic importance for the outcome of interest. For adverse pregnancy outcomes, two multivariable regression models were analyzed; model 1 only includes maternal age and BMI, whereas model 2 includes maternal age, BMI and other variables that exhibit a *P*-value of < 0.05 in the univariate analysis. The number of oocytes retrieved was not putted in the regression model as it is highly correlated with both serum E_2_ level and number of fertilized occytes (**Supplemental Table 1**). All *P* values are two sided and statistical significance was established as *P*<0.05. All analyses were conducted using SPSS (version 23.0; SPSS Inc.).

## Results

### Population Characteristics

A total of 7,678 couples undergoing their first IVF treatments between 2010 and 2017 were included in the cohort, with 666 women diagnosed with PCOS and 7,012 controls. The prevalence of PCOS was 8.7% in this study population. The predominant PCOS phenotype in this study was oligo-menorrhea with polycystic ovaries; 85.7% of the PCOS population displayed oligo- or amenorrhea, 61.4% had hyperandrogenism, 78.7% showed polycystic ovary morphology, and 25.8% met all three criteria. The range of BMI in PCOS women in this study was 14.88-31.25 kg/m^2^. There were 23.3% (155/666) PCOS women with BMI > 25 kg/m^2^ and 76.7% (511/666) PCOS women with BMI ≤ 25 kg/m^2^.

The women with PCOS were slightly younger (30.0 vs. 31.0 years; *P*<0.001), had a higher BMI (22.3 vs. 20.7 kg/m^2^; *P*<0.001), and longer infertility duration (3.8 vs. 3.7 years; *P*<0.001) compared with the control group (**Table 1**). The PCOS population was more frequently diagnosed with primary infertility (66.8% vs. 51.2%; *P*<0.001). More ovulatory disorders (73.4% vs. 0.5%; *P*<0.001), higher baseline LH level (5.8 vs. 3.6 IU/L; *P*<0.001) and lower FSH level (5.1 vs. 5.6 IU/L; *P*<0.001) were found in the PCOS group compared with the control group. Women with PCOS had significantly higher total T level (0.5 vs. 0.3 ng/ml, *P*<0.001) than women without PCOS. Fasting glucose (5.2 vs. 5.1 mmol/L, *P*<0.001) and fasting insulin (10.7 vs. 9.5 μU/ml, *P*<0.001) were significantly higher in PCOS group. QUICKI was significantly lower in women with PCOS compared with women without PCOS (0.333 vs. 0.340, *P*=0.004). TSH did not differ between the two groups (*P*=0.192).

**Table 1:** Clinical characteristics of women with polycystic ovary syndrome (PCOS) and controls during their first IVF treatment cycle.

Maternal characteristics	PCOS (n=666)	Control (n=7012)	*P*-value
**Maternal age (y)**	30.0 (27.0–32.0)	31.0 (29.0–34.0)	<0.001
**Maternal BMI (kg/m^2^)**	22.3 (20.3–25.0)	20.7 (19.2–22.6)	<0.001
**Type of infertility**			<0.001
Primary	445 (66.8%)	3,587 (51.2%)	
Secondary	221 (33.2%)	3,425 (48.8%)	
**Infertility duration (y)**	3.8 ± 2.2	3.7 ± 2.6	<0.001
**Infertility factors**			
Uterine and tubal factor	50 (7.5%)	3,375 (48.1%)	<0.001
Ovulatory disorders	489 (73.4%)	38 (0.5%)	<0.001
Endometriosis	15 (2.3%)	510 (7.3%)	<0.001
Male factor	8 (1.2%)	888 (12.7%)	<0.001
Female and male factor	94 (14.1%)	559 (8.0%)	<0.001
Unexplained	10 (1.5%)	1,642 (23.4%)	<0.001
**Baseline FSH level (IU/L)**	5.1 (4.3–6.0)	5.6 (4.7–6.8)	<0.001
**Baseline LH level (IU/L)**	5.8 (3.8–9.0)	3.6 (2.7–5.0)	<0.001
**Total T (ng/ml)**	0.5 (0.4–0.8)	0.3 (0.2–0.4)	<0.001
**TSH (μIU/ml)**	1.8 (1.3–2.5)	1.9 (1.3–2.6)	0.192
**Fasting glucose (mmol/L)**	5.2 (4.9–5.5)	5.1 (4.8–5.5)	<0.001
**Fasting insulin (μU/ml)**	10.7 (7.6–16.5)	9.5 (6.5–13.3)	<0.001
**QUICKI**	0.333 ± 0.029	0.340 ± 0.030	0.004
**Duration of gonadotropin stimulation (d)**	10.0 (9.0–12.0)	9.0 (8.0–10.0)	<0.001
**Total dose of gonadotropin (IU)**	1,650.0 (1,350.0–2,175.0)	2,400.0 (1,800.0–3,000.0)	<0.001
**Serum E_2_ level (pg/ml) on hCG day**	2,602.0 (1,747.0–3,740.8)	2,495.0 (1,734.3–3,430.0)	0.040
**EMT (mm) on hCG day**	11.0 (10.0–13.0)	11.0 (10.0−13.0)	0.737
**No. of oocyte retrieved**	15.0 (12.0–19.0)	13.0 (9.0−17.0)	<0.001
**No. of fertilized occytes**	12.0 (10.0–16.0)	10.0 (7.0–14.0)	<0.001
**No. of embryos transferred**	2.2 ± 0.5	2.4 ± 0.7	<0.001
**Cycles with different technologies**			0.003
IVF	536 (80.5%)	5,281 (75.3%)	
ICSI	130 (19.5%)	1,731 (24.7%)	
**Embryo type**			0.430
Cleavage embryo	609 (91.4%)	6,472 (92.3%)	
Blastocyst	57 (8.6%)	540 (7.7%)	
**Embryo quality**			0.002
Cycle with high-quality embryos	650 (97.6%)	6,658 (95.0%)	
Cycles without high-quality embryos	16 (2.4%)	354 (5.0%)	

BMI, body mass index; QUICKI, quantitative insulin sensitivity check index; E_2_, estradiol; EMT, endometrial thickness; IVF, in vitro fertilization; ICSI, intracytoplasmic sperm injection; hCG, human chorionic gonadotropin.

Values are numbers (percentages) of cases, mean ± standard deviation or median (interquartile range).

Continuous variables: Mann-Whitney U-test or independent t-test; categorical variables: Chi-square test or Fisher’s exact test.

Compared with the control group, longer duration of gonadotropin stimulation (10.0 vs. 9.0 days; *P*<0.001), less gonadotropin was used (1650.0 vs. 2400.0 IU; *P*<0.001) and higher serum E_2_ levels on the day of hCG trigger (2602.0 vs. 2495.0 pg/ml; *P*=0.040) were noticed in the PCOS group. There was no significant difference in endometrial thickness before embryo transfer between these two groups (*P*=0.737).

In the present study, PCOS women had a trend toward more oocytes retrieved (15.0 vs. 13.0; *P*<0.001) and higher number of fertilization (12.0 vs. 10.0; *P*<0.001), as well as fewer transferred embryos (2.2 vs. 2.4; *P*<0.001). A higher frequency of IVF (80.5% vs. 75.3%; *P*=0.003) and more cycles with high-quality embryos were found in PCOS group compared with the control group (97.6% vs. 95.0%; *P*=0.002). Embryo type (cleavage-stage embryo or blastocyst) did not differ significantly between the two groups (*P*=0.430).

### Pregnancy Outcomes

The implantation rate, clinical pregnancy rate and live birth rate were significantly higher in the PCOS group compared with the control group (49.3% vs. 38.1%, 70.9% vs. 59.8%, 58.3% vs. 52.1%, respectively; *P*<0.001, *P*<0.001, *P*=0.002, respectively, see **Table 2**). Nevertheless, the delivery type (cesarean or eutocia) exhibited no difference between these two groups (*P*=0.967). As for the adverse pregnancy outcomes, miscarriage and multiple pregnancies were more common among women with PCOS diagnosis than among those without (16.5% vs. 11.2%, 42.0% vs. 35.8%, respectively; *P*=0.001, *P*=0.016, respectively). In addition, women with PCOS were more prone to deliver preterm (<37 weeks) than the control group (26.5% vs. 21.9%; *P*=0.039). The very preterm delivery rate (<32 weeks) was also significantly higher in the PCOS group (3.9% vs. 1.6%; *P*=0.002). PIH were more often reported in the PCOS group (3.9% vs. 2.2%; *P*=0.031). However, the morbidity of GDM was similar in both groups (*P*=0.665).

**Table 2:** Pregnancy outcomes and complications of women with polycystic ovary syndrome (PCOS) diagnosis and controls during their first *in vitro* fertilization (IVF) treatment cycle.

Outcomes	PCOS (n=666)	Control (n=7,012)	*P*-value
**Implantation rate**	49.3% (732/1,484)	38.1% (6,290/16,521)	<0.001
**Clinical pregnancy rate**	70.9% (472/666)	59.8% (4,190/7,012)	<0.001
**Miscarriage rate**	16.5% (78/472)	11.2% (469/4,190)	0.001
Early miscarriage rate	9.1% (43/472)	7.8% (326/4,190)	0.310
Late miscarriage rate	7.4% (35/472)	3.4% (143/4,190)	<0.001
**Live birth rate**	58.3% (381/653)	52.1% (3,584/6,874)	0.002
**Term delivery rate**	73.5% (280/381)	78.1% (2,800/3,584)	0.039
**Preterm delivery rate**	26.5% (101/381)	21.9% (784/3,584)	0.039
≥ 34 and <37 weeks	19.2% (73/381)	17.9% (641/3,584)	0.538
≥ 32 and <34 weeks	3.4% (13/381)	2.3% (84/3,584)	0.199
< 32 weeks weeks	3.9% (15/381)	1.6% (59/3,584)	0.002
**Delivery type**			0.967
Cesarean	26.8% (102/381)	26.9% (963/3,584)	
Eutocia	73.2% (279/381)	73.1% (2,621/3,584)	
**No. of live babies delivered**			0.016
1	58.0% (221/381)	64.2% (2,302/3,584)	
≥2	42.0% (160/381)	35.8% (1,282/3,584)	
**Pregnancy complications**			
Gestational diabetes mellitus	9.7% (37/381)	9.0% (324/3,584)	0.665
Pregnancy-induced hypertension	3.9% (15/381)	2.2% (78/3,584)	0.031

Categorical variables are presented as percentages (%) and analyzed by Chi-square test or Fisher’s exact test.

Logistic regression analysis was performed to analyze the association between PCOS and the pregnancy outcomes (**Tables 3**, **4** and **Supplemental Table 2**). As indicated in **Table 3**, more live births were positively correlated with a PCOS diagnosis, younger age, shorter duration of infertility, less gonadotropin used, higher E_2_ levels and thicker endometrial thickness on the day of hCG trigger, more oocytes retrieved and fertilized, fewer embryos transferred, but more blastocysts and higher-quality embryos transferred. After adjusted for maternal age and BMI, women with PCOS still had a higher live birth rate (aOR 1.213, 95% CI 1.027–1.433, *P*=0.023). However, after correcting for the effects of all the above-mentioned confounders, a PCOS diagnosis was no longer associated with a higher live birth rate (aOR 1.012, 95% CI 0.852–1.204, *P*=0.888). As for clinical pregnancy outcome, when these factors were adjusted for, having a PCOS diagnosis remained associated with a higher clinical pregnancy rate (aOR 1.248, 95% CI 1.038^–^1.501, *P*=0.019).

**Table 3:** Logistic regression analysis on the contribution of the potential predicting variables to live birth and clinical pregnancy outcomes.

Variables	Live birth						Clinical pregnancy					
	OR (95% CI)	*P*-value	Adjusted OR(95% CI)^a^	*P*-value^a^	Adjusted OR(95% CI)^b^	*P*-value^b^	OR (95% CI)	*P*-value	Adjusted OR(95% CI)^a^	*P*-value^a^	Adjusted OR(95% CI)^b^	*P*-value^b^
**PCOS diagnosis**	1.287 (1.093−1.514)	0.002	1.213 (1.027−1.433)	0.023	1.012 (0.852−1.204)	0.888	1.639 (1.377−1.950)	<0.001	1.532 (1.284−1.830)	<0.001	1.248 (1.038−1.501)	0.019
**Maternal age (y)**	0.959 (0.947−0.971)	<0.001	0.960 (0.948−0.972)	<0.001	0.983 (0.968−0.997)	0.021	0.971 (0.959−0.984)	<0.001	0.972 (0.960−0.985)	<0.001	0.993 (0.978−1.008)	0.333
**Maternal BMI (kg/m^2^)**	0.999 (0.983−1.016)	0.946	1.004 (0.987−1.021)	0.677	0.996 (0.979−1.014)	0.667	1.021 (1.004−1.038)	0.014	1.020 (1.002−1.037)	0.026	1.008 (0.990−1.027)	0.362
**Infertility duration (y)**	0.974 (0.957−0.991)	0.003			0.989 (0.971−1.008)	0.271	0.986 (0.969−1.004)	0.126			0.996 (0.977−1.016)	0.707
**Total dose of gonadotropin (IU)^1^**	0.718 (0.679−0.759)	<0.001			0.798 (0.750−0.850)	<0.001	0.682 (0.645−0.722)	<0.001			0.753 (0.707−0.802)	<0.001
**Serum E_2_ level (pg/ml) on hCG day^2^**	1.057 (1.028−1.087)	<0.001			0.968 (0.936−1.001)	0.058	1.045 (1.016−1.075)	0.002			0.939 (0.907−0.972)	<0.001
**EMT (mm) on hCG day**	1.087 (1.066−1.109)	<0.001			1.091 (1.069−1.114)	<0.001	1.087 (1.065−1.109)	<0.001			1.092 (1.070−1.115)	<0.001
**No. of fertilized occytes**	1.050 (1.041−1.060)	<0.001			1.030 (1.018−1.041)	<0.001	1.057 (1.047−1.067)	<0.001			1.037 (1.025−1.049)	<0.001
**No. of embryos transferred**	0.880 (0.821−0.943)	<0.001			1.009 (0.930−1.095)	0.830	0.952 (0.888−1.021)	0.169			1.115 (1.026−1.211)	0.010
**Embryo type**												
Cleavage embryo	Ref	Ref			Ref	Ref	Ref	Ref			Ref	Ref
Blastocyst	2.005 (1.667−2.410)	<0.001			1.951 (1.603−2.376)	<0.001	2.126 (1.753−2.578)	<0.001			2.210 (1.797−2.719)	<0.001
**Embryo quality**												
Cycle with high-quality embryos	Ref	Ref			Ref	Ref	Ref	Ref			Ref	Ref
Cycles without high-quality embryos	0.209 (0.161−0.272)	<0.001			0.257 (0.197−0.336)	<0.001	0.210 (0.166−0.267)	<0.001			0.270 (0.211−0.345)	<0.001

All the variables inputted in the model were shown in **Table 3**. BMI, body mass index; CI, confidence interval; OR, odds ratio; E_2_,estradiol; hCG, human chorionic gonadotropin; EMT, endometrial thickness; Ref, reference; ^1^per 1,000 IU increased; ^2^per 1,000 pg/ml increased.

a Adjusted for maternal age and BMI.

b Adjusted for maternal age, BMI, infertility duration, total dose of gonadotropin, serum E_2_ level and endometrial thickness on hCG day, number of fertilized occytes, number of embryos transferred, embryo type and embryo quality.

**Table 4:** Univariable and multivariable regression analysis of risk of miscarriage, preterm delivery and pregnancy-induced hypertension.

Outcomes	Variables	OR (95% CI)	*P*-value	Adjusted OR(95% CI)^a^	*P*-value^a^	Adjusted OR(95% CI)^b^	*P*-value^b^
**Miscarriage**	**PCOS diagnosis**	1.571 (1.210−2.039)	0.001	1.585 (1.210−2.078)	0.001	1.629 (1.240−2.141)	<0.001
	**Maternal age (y)**	1.070 (1.043−1.098)	<0.001	1.069 (1.042−1.098)	<0.001	1.037 (1.007−1.067)	0.015
	**Maternal BMI (kg/m^2^)**	1.072 (1.040−1.106)	<0.001	1.051 (1.018−1.086)	0.002	1.046 (1.012−1.080)	0.008
	**Infertility duration (y)**	1.054 (1.019−1.089)	0.002			1.021 (0.986−1.057)	0.237
	**Serum E_2_ level (pg/ml) on hCG day^2^**	0.925 (0.871−0.982)	0.010			0.945 (0.890−1.004)	0.069
	**EMT (mm) on hCG day**	0.953 (0.917−0.991)	0.015			0.949 (0.912−0.987)	0.009
	**No. of embryos transferred**	1.473 (1.296−1.674)	<0.001			1.348 (1.160−1.565)	<0.001
	**Embryo type**						
	Cleavage embryo	Ref	Ref			Ref	Ref
	Blastocyst	0.566 (0.393−0.817)	0.002			0.693 (0.472−1.017)	0.061
	**Embryo quality**						
	Cycle with high-quality embryos	Ref	Ref			Ref	Ref
	Cycles without high-quality embryos	1.887 (1.133−3.143)	0.015			1.846 (1.098−3.104)	0.021
**Preterm delivery (**< **37 weeks)**	**PCOS diagnosis**	1.288 (1.012−1.639)	0.040	1.236 (0.966−1.582)	0.092	1.187 (0.922−1.528)	0.185
	**Maternal age (y)**	0.985 (0.964−1.006)	0.158	0.985 (0.964−1.007)	0.172	0.969 (0.946−0.992)	0.009
	**Maternal BMI (kg/m^2^)**	1.016 (0.989−1.044)	0.243	1.015 (0.987−1.044)	0.285	1.015 (0.986−1.044)	0.318
	**Total dose of gonadotropin (IU)^1^**	0.903 (0.821−0.994)	0.037			0.933 (0.841−1.036)	0.194
	**No. of fertilized occytes**	1.017 (1.002−1.032)	0.023			1.010 (0.994−1.026)	0.215
	**No. of embryos transferred**	1.209 (1.075−1.359)	0.002			1.428 (1.252−1.628)	<0.001
	**Embryo type**						
	Cleavage embryo	Ref	Ref			Ref	Ref
	Blastocyst	1.622 (1.285−2.047)	<0.001			1.964 (1.534−2.514)	<0.001
	**Pregnancy-induced hypertension**	2.468 (1.621−3.757)	<0.001			2.439 (1.595−3.731)	<0.001
**Preterm delivery (**< **32 weeks)**	**PCOS diagnosis**	2.448 (1.375−4.358)	0.002	2.135 (1.170−3.895)	0.013	2.072 (1.133−3.791)	0.018
	**Maternal age (y)**	0.934 (1.003−1.071)	0.934	1.002 (0.937−1.072)	0.948	1.000 (0.935−1.069)	0.997
	**Maternal BMI (kg/m^2^)**	1.114 (1.032−1.204)	0.006	1.094 (1.010−1.184)	0.028	1.089 (1.006−1.179)	0.035
	**Pregnancy-induced hypertension**	3.132 (1.233−7.953)	0.016			2.727 (1.059−7.020)	0.038
**Pregnancy-induced hypertension**	**PCOS diagnosis**	1.842 (1.049−3.234)	0.034	1.784 (0.996−3.197)	0.052	1.764 (0.981−3.171)	0.058
	**Maternal age (y)**	1.042 (0.982−1.106)	0.171	1.043 (0.981−1.108)	0.177	1.017 (0.955−1.084)	0.595
	**Maternal BMI (kg/m^2^)**	1.077 (1.003−1.156)	0.040	1.057 (0.982−1.137)	0.143	1.032 (0.958−1.111)	0.412
	**Infertility duration (y)**	1.098 (1.023−1.178)	0.010			1.081 (1.003−1.165)	0.042
	**Serum E_2_ level (pg/ml) on hCG day^2^**	0.803 (0.683−0.945)	0.008			0.824 (0.700−0.969)	0.020

All the variables inputted in the model were shown in **Table 4**. BMI, body mass index; CI, confidence interval; OR, odds ratio; E_2_, estradiol; hCG, human chorionic gonadotropin; EMT, endometrial thickness; Ref, reference; ^1^per 1,000 IU increased; ^2^per 1,000 pg/ml increased.

a Adjusted for maternal age and BMI.

b Adjusted for maternal age, BMI, and other variables based on the univariate logistic regression model.

Multiple logistic regression showed that the PCOS diagnosis, maternal age, BMI, duration of infertility, serum E_2_ level and endometrial thickness on the day of hCG trigger, number of embryos transferred, embryo type and quality were all significantly correlated with miscarriage on univariate analysis (**Table 4**). After controlling for maternal age and BMI, miscarriage was still positively correlated with a PCOS diagnosis (aOR 1.585, 95% CI 1.210–2.078, *P*=0.001). Moreover, a secondary analysis was performed including all the above-mentioned factors in the model, which did not substantially alter the estimates, PCOS remained positively associated with an increased risk of miscarriage (aOR 1.629, 95% CI 1.240–2.141, *P*<0.001).

In another multiple regression analysis (correcting for the effects of a PCOS diagnosis, age, BMI, number of embryos transferred, embryo type and PIH), younger maternal age, more embryo transferred, more blastocysts and the development of PIH (aOR 2.439, 95% CI 1.595–3.731, *P*<0.001) were associated with an increased risk of preterm delivery before 37 weeks of gestation. However, a PCOS diagnosis was not associated with preterm delivery before 37 weeks of gestation in this analysis (aOR 1.187, 95% CI 0.922–1.528, *P*=0.185). For the very preterm delivery (<32 weeks), after adjust for maternal age, BMI and PIH (since no other confounders were found according to the univariate logistic regression model), a PCOS diagnosis (aOR 2.072, 95% CI 1.133–3.791, *P*=0.018), higher BMI and the development of PIH (aOR 2.727, 95% CI 1.059–7.020, *P*=0.038) remained associated with an increased risk of preterm delivery before 32 weeks of gestation.

PCOS was no longer associated with increased risk of PIH after adjusting for age, BMI, duration of infertility and serum E_2_ level (aOR 1.764, 95% CI 0.981–3.171, *P*=0.058). Moreover, we also performed a stratified analysis for these adverse pregnancy outcomes among women overweight/obese or lean defined by a BMI over 25 kg/m^2^ and those with a BMI less than 25 kg/m^2^ (**Supplemental Table 3**).

Logistic regression analysis was also performed to analyze the association between insulin resistance and the pregnancy outcomes in PCOS women (**Supplemental Table 4**). QUICKI was significantly correlated with miscarriage and live birth on univariate analysis. After controlling for maternal age, QUICKI remained associated with a lower miscarriage rate (aOR 0.816, 95% CI 0.703–0.947, *P*=0.007) and a higher live birth rate (aOR 1.144, 95% CI 1.038–1.262, *P*=0.007). However, QUICKI was not associated with clinical pregnancy and GDM in this analysis.

## Discussion

In the present study, we found that women with PCOS had higher live birth rate, clinical pregnancy rate and implantation rate, as well as an increased risk of pregnancy complications (miscarriage, preterm delivery and PIH) in their first IVF treatment when compared with non-PCOS controls. Among all these outcomes, higher clinical pregnancy rate, implantation rate, miscarriage rate, and very preterm delivery rate (<32weeks) were still maintained in the PCOS population after adjusting for the following risk factors: maternal age, BMI, infertility duration, total dose of gonadotropin, serum E_2_ level and endometrial thickness on the day of hCG trigger, number of fertilized occytes, number of embryos transferred, embryo type and quality.

We found women with PCOS had significantly greater numbers of oocytes retrieved and fertilized. We postulated that by retrieving more oocytes and achieving higher fertilization number, there could be more embryo selection, and therefore higher subsequent pregnancy rate. Moreover, a significantly increased live birth rate was observed in women with PCOS even after correcting for maternal age and BMI, however, this effect faded when adjusted for all the above-mentioned risk factors.

It is still under debate whether women with PCOS have an increased risk of miscarriage compared with controls. Although available data show conflicting results, miscarriage rate was suggested to be comparable based on the PCOS consensus of 2012 (1). One meta-analysis showed no difference in miscarriage rate between women with PCOS and those without undergoing IVF (12). Our data showed a significantly increased risk of miscarriage among PCOS subjects compared with controls, which is consistent to a large Australian study also demonstrating that the miscarriage rate was more frequent in women with PCOS than in controls (20% vs. 15%; *P*=0.003) (13). Furthermore, in our study, the difference was still statistically significant after adjusting for important confounders. On subgroup analysis, the association between PCOS and miscarriage was only seen among the lean population after adjusting for age and BMI (aOR 1.599, 95% CI 1.173–2.181, *P*=0.003). Our results indicate that there may be other intrinsic “PCOS factors” which may contribute to miscarriage except BMI. A recent study shows that PCOS-induced miscarriage may be associated with hyperandrogenism and insulin resistance, which could disrupt normal mitochondrial function and homeostasis and a resulting imbalance between oxidative and antioxidative stress responses in the gravid uterus (14). However, this is an *in vivo* animal study, and the relevance of the results for humans remains to be established.

Two meta-analyses demonstrated that women with PCOS have a 2-fold increased risk of preterm delivery (6, 7), whereas another meta-analysis demonstrated no effect (8). In a large Swedish study, infants born to mothers with PCOS were more frequently delivered prematurely (OR 2.21, 95% CI 1.69–2.90) (15). Another cohort study confirmed an increased risk of preterm delivery (OR 2.02, 95% CI 1.13–3.61) (16). Here, we observed an increased rate of preterm delivery before 32 weeks among PCOS subjects, but no difference in the rate of preterm delivery before 37 weeks after adjusting for the confounders. However, we found that the development of PIH was associated with preterm delivery both before 37 weeks and before 32 weeks, suggesting that preterm delivery may occur through development of PIH. On subgroup analysis, there was a strong correlation between PCOS and preterm delivery before 37 weeks (aOR 1.360, 95% CI 1.035–1.787, *P*=0.027) among lean population. This finding was consistent with the previous study, which also showed that a significantly increased risk in preterm delivery was only found among the lean PCOS subjects (17). Hyperandrogenism, disturbed glucose metabolism and higher levels of inflammatory markers have been regarded as potential causes of preterm delivery in women with PCOS (16, 18). Hyperandrogenism and insulin resistance characteristic of PCOS are associated with disturbances in the coagulation and fibrinolytic system, resulting in endothelial dysfunction, atherothrombosis and chronic low grade inflammation, which may lead to micro-vasculopathy and placental dysfunction (16). Several studies have reported that the inflammatory cytokine levels were higher in amniotic fluid of women in premature labor (19, 20). Thus, underlying inflammatory mediators associated with PCOS may also contribute to predisposition for preterm delivery. Additionally, a recent study showed that higher antimüllerian hormone (AMH) levels were a risk factor for preterm delivery in women with PCOS (21). This result may be explained by the higher AMH level observed in PCOS women during pregnancy was considered to have an impact on the endocrine system of the fetus and offspring (22). Taken together, we demonstrate an increased risk of preterm delivery in women with PCOS compared with women without PCOS. Thus, PCOS women during pregnancy might need increased attention and closer follow-up to improve the outcome.

The higher rate of PIH (3.9%) in women with PCOS in our study is similar with those in the literature (3%–32%) (7). However, this may be an underestimate as patients may have unreported or underreported symptoms, or they may have been admitted to a different hospital. The mechanisms responsible for hypertension in women with PCOS may be explained as follows: first, insulin resistance could cause secondary hyperinsulinemia, which may produce enhanced sodium retention and play a role in the development of hypertension (23, 24); second, insulin could stimulate the release of insulin-like growth factor (IGF-1), which may determine the vascular smooth muscle hypertrophy and thus contribute to the hypertension (25); third, hyperandrogenemia also seems to be related to blood pressure (15). There have been three meta-analyses reported a three to four times increased risk of PIH in women with PCOS (6–8). Similarly, we observed increased rate of PIH in the PCOS population. However, after adjusting for the confounders, the prevalence of PIH among women with PCOS was somewhat higher but only borderline significant (aOR 1.764, 95% CI 0.981–3.171, *P*=0.058), suggesting that there may be other risk factors contributing to PIH. On subgroup analysis, no significant different on the morbidity of PIH was seen among both lean and overweight/obese PCOS women.

GDM is the most commonly described pregnancy complication in women with PCOS. The early diagnosis and treatment could significantly reduce the incidence and severity of related maternal and neonatal complications (26). Higher GDM rate in PCOS women was thought to be due to insufficient pancreatic β-cell function to overcome the placental hormone-mediated exacerbation of pre-existing insulin resistance during pregnancy (27). Three meta-analyses reported a three times higher risk of GDM in women with PCOS (6–8). Among all the studies included, one Swedish population-based cohort study compared 3787 women with PCOS and 1191336 women without PCOS and showed than GDM was more than 2-fold higher in women with PCOS after adjusting data for confounders (OR 2.32, 95% CI 1.88–2.88) (15). However, the women they included had more severe disease as exposed, and the findings may consequently not be generalizable to all women with PCOS. In this study, we observed the prevalence of GDM was 9.7% among PCOS subjects, which is consistent with the reported rates (3%–40%) (28). However, we failed to find a statistically significant difference in the risk of GDM between women with PCOS and controls. This result may be partially explained by the relatively young population in our study, as many studies have indicated that advanced maternal age is an important risk factor for GDM (29, 30).

Insulin resistance appears to be a crucial aetiological characteristic in most women with PCOS (31). Insulin resistance and compensatory hyperinsulinaemia could increase ovarian androgen production and reduce hepatic sex-hormone binding globulin (SHBG) production, resulting in androgen excess (31). Both androgen excess and insulin resistance underpin the features of PCOS. In this study, we used QUICKI as a surrogate marker for insulin resistance and found that it was significantly correlated with miscarriage and live birth after controlling for maternal age. It suggests that insulin resistance is an important factor behind the increased risk of adverse pregnancy outcomes in women with PCOS. However, QUICKI was not associated with GDM in our study. The results may be explained by the administration of metformin among PCOS women, which appears to be an inhibition of hepatic glucose production and an increase in peripheral insulin sensitivity (32).

Multiple pregnancies are considered to be one of the most important adverse outcomes in patients following assisted reproductive technologies. Negative pregnancy complications associated with multiple gestations have been well documented, including increased risk of PIH, pre-eclampsia, preterm delivery, neonatal mortality and cesarean delivery (33, 34). The higher incidence of multiple pregnancies among PCOS patients in our study may therefore underlie the poorer observed outcomes. Moreover, although several studies have suggested that women with PCOS showed a higher rate of cesarean section, this was not observed in our study (6–8, 35).

Increasing evidences have showed a higher rate of pregnancy complications in women with PCOS. The increased incidence can partially be explained by higher BMI in women with PCOS. However, after adjusted for BMI, there seems to be an intrinsic “PCOS factors” which may contribute to pregnancy complications. The pathophysiology and intrinsic mechanisms underlying PCOS are complex and considerably intertwined. The interplay between these mechanisms results in the clinical features of PCOS, including hyperandrogenism, PCOM and ovulatory dysfunction. Insulin resistance in PCOS is an intrinsic characteristic, which is aggravated by obesity but not simply a consequence of obesity. Insulin resistance and compensatory hyperinsulinemia adversely effects ovarian androgen production in PCOS (36). Studies have shown that adipocytes and adipocyte function are aberrant in PCOS, favoring insulin resistance and subclinical inflammation (37). Inflammatory cytokines could suppress insulin-mediated glucose transport to a greater degree in adipocytes derived from patients with PCOS (38). Overall, primary defects in adipocyte functioning, including insulin-mediated glucose transport (39), GLUT4 production (40), and insulin-stimulated inhibition of lipolysis (41) have been reported in PCOS. In addition to decreased hepatic synthesis of SHBG caused by hyperinsulinaemia, hyperandrogenism could also be resulted from increased expression of several genes encoding steroidogenic enzymes in follicular theca cells, like *DENND1A* and *CYP17A1* (42). Hyperandrogenism and insulin resistance characteristic of PCOS may also play a crucial role during trophoblast invasion and placentation, and thus increase the long-term risk for mothers and children (16).

In our study, women with PCOS were younger, and had higher BMI compared with women without PCOS which could be perceived as nonideal controls. Although matching does not offer advantage over independent control selection with regard to study validity under case-control design, it could improve study precision (43). Thus, we also performed a stratified analysis for the adverse pregnancy outcomes among women overweight/obese or lean. Regarding the difference in maternal age, it is minimal (30.0 vs. 31.0 years; *P*<0.001) and the difference is probably due to the large number of subjects involved. An important strength of this study is the large size of the cohort, which enabled us to include more relevant confounders to provide a more precise estimation of the risks of pregnancy complications in women with PCOS than in former studies. Additionally, we were able to distinguish between preterm and very preterm delivery, which is important as the relationship between PCOS and preterm delivery might differ according to gestational age. Moreover, subgroup analysis was performed to stratify risk for the pregnancy complications by lean vs. overweight/obese PCOS, which makes it possible to analyze the relationship between PCOS and adverse pregnancy outcomes under different subgroups. Our study also has a number of limitations. First, potential selection bias could have occurred since the infertility factors were different between PCOS and non-PCOS patients. Second, our study relied on self-reported data of pregnancy complications, which may lead to an underestimation of the prevalence of PIH and GDM. Third, the retrospective nature made it difficult to study several pregnancy and neonatal outcomes, such as antepartum hemorrhage, pre-eclampsia, birthweight, small or large for gestational age neonates. Furthermore, we were unable to stratify preterm delivery for cause (e.g. premature ruptures of membranes, cervical insufficiency) or spontaneous versus induced preterm delivery, or analyze between PIH and pre-eclampsia. Finally, the results of this study cannot be generalized to all IVF cases since we only analyzed fresh embryo transfer cycles.

## Conclusions

In conclusion, PCOS was associated with an increased risk of several pregnancy complications, such as miscarriage and preterm delivery, even after adjustments for all of those potential confounders. Although further multi-center prospective studies are required to validate our findings, our results indicate that women with PCOS undergoing IVF may need increased surveillance during pregnancy and parturition. Furthermore, pregnancy complications may influence long-term maternal health, such as cardiovascular disease and diabetes (44, 45). Thus, a better understanding of pregnancy complication rates among PCOS women will reduce the risk of both obstetric and neonatal complications and improve overall management of those patients.

## Data Availability Statement

The raw data supporting the conclusions of this article will be made available by the authors, without undue reservation.

## Ethics Statement

The studies involving human participants were reviewed and approved by The Reproductive Research Ethics Committees of Shenzhen Zhongshan Urology Hospital. Written informed consent for participation was not required for this study in accordance with the national legislation and the institutional requirements.

## Author Contributions

SL, LD, and YZ are responsible for the concept and the study design. LL and LW performed the data collection, and SL did the statistical analysis. SL drafted the manuscript. MM, SX, XH, LH, RL, and CH contributed to the critical discussion, interpretation and editing of the manuscript.

## Funding

This work was supported by Natural Science Foundation of Guangdong Province (2019A1515010914), Sanming Project of Medicine in Shenzhen (SZSM201502035), National Natural Science Foundation of China (No. 81701529), Special Funds for Science, Technology Development of Guangdong Province (2017A020214006) and National Key Research & Developmental Program of China (2018YFC1003900).

## Conflict of Interest

The authors declare that the research was conducted in the absence of any commercial or financial relationships that could be construed as a potential conflict of interest.

## Supplementary Material

Supplementary Figure 1 Flow chart showing the identification of the study population.
