# Supplementary figures and images for: Pregnancy Outcomes of Women With Polycystic Ovary Syndrome for the First In Vitro Fertilization Treatment: A Retrospective Cohort Study With 7678 Patients
Source: Front Endocrinol (Lausanne). 2020 Sep 25;11:575337. doi: 10.3389/fendo.2020.575337 (PMC7546360; doi:10.3389/fendo.2020.575337)

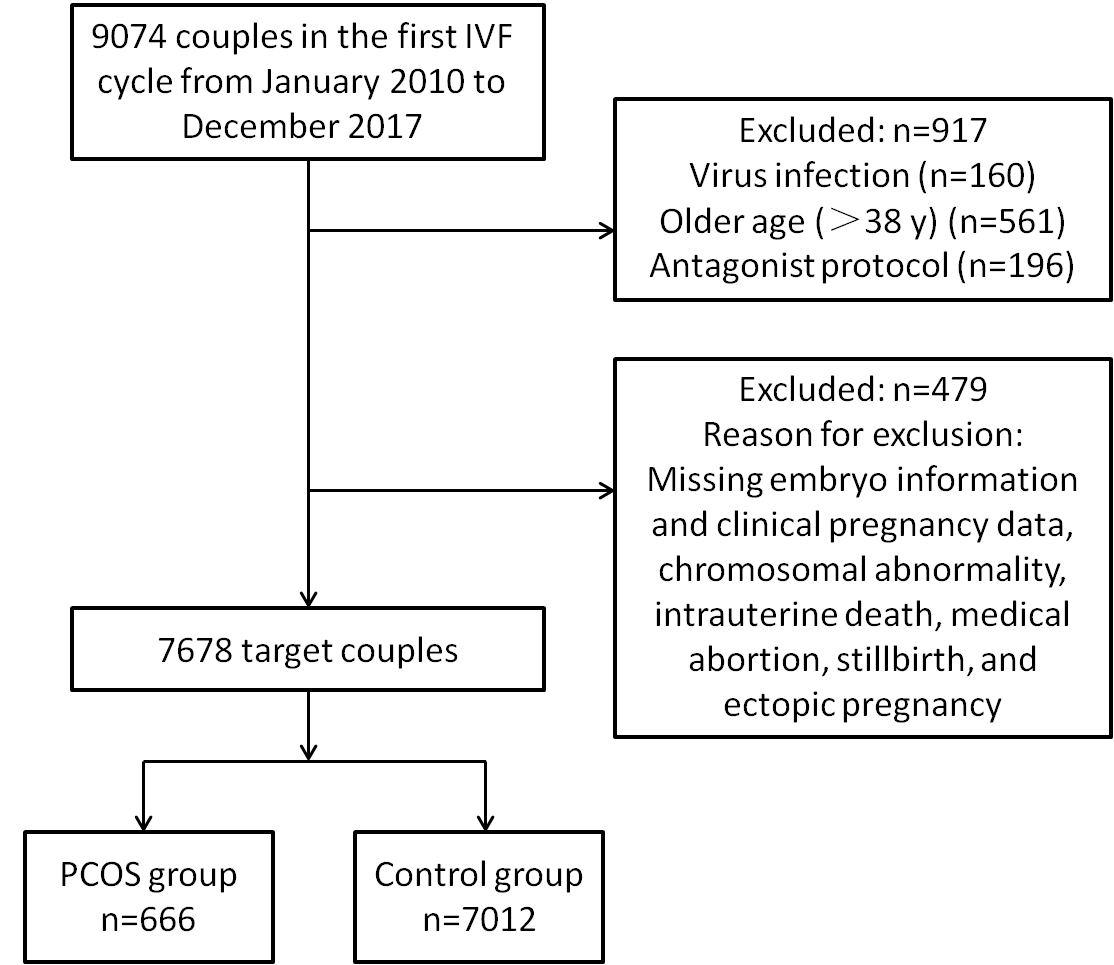

Supplement: Supplementary Figure 1 — Flow chart showing the identification of the study population. [file Image_1.tif]
