# Supplementary material for: Pregnancy Outcomes of Women With Polycystic Ovary Syndrome for the First In Vitro Fertilization Treatment: A Retrospective Cohort Study With 7678 Patients
Source: Front Endocrinol (Lausanne). 2020 Sep 25;11:575337. doi: 10.3389/fendo.2020.575337 (PMC7546360; doi:10.3389/fendo.2020.575337)
Supplement: Supplementary file 2 [file Table_1.docx]

**SUPPLEMENTAL TABLE 1.**

Correlations between factors in the logistic models.

|  | | PCOS diagnosis | Maternal age | Infertility duration | Maternal BMI | Infertility factor | EMT | GN dose (IU) | E_2_ on hCG day | No. of oocyte retrieved | No. of fertilized occytes | Embryo type | Embryo quality | No. of embryo transferred |
| --- | --- | --- | --- | --- | --- | --- | --- | --- | --- | --- | --- | --- | --- | --- |
| PCOS diagnosis | Pearson correlation | 1 | -.104^**^ | .018 | .162^**^ | -.077^**^ | .006 | -.180^**^ | .031^**^ | .156^**^ | .148^**^ | .009 | -.035^**^ | -.055^**^ |
|  | Sig. (2-tailed) |  | .000 | .122 | .000 | .000 | .630 | .000 | .006 | .000 | .000 | .430 | .002 | .000 |
|  | No. | 7678 | 7678 | 7678 | 7678 | 7678 | 7678 | 7678 | 7678 | 7678 | 7678 | 7678 | 7678 | 7678 |
| Maternal age | Pearson correlation | -.104^**^ | 1 | .248^**^ | .138^**^ | .041^**^ | -.059^**^ | .253^**^ | -.127^**^ | -.232^**^ | -.207^**^ | .017 | .032^**^ | .327^**^ |
|  | Sig. (2-tailed) | .000 |  | .000 | .000 | .000 | .000 | .000 | .000 | .000 | .000 | .138 | .005 | .000 |
|  | No. | 7678 | 7678 | 7678 | 7678 | 7678 | 7678 | 7678 | 7678 | 7678 | 7678 | 7678 | 7678 | 7678 |
| Infertility duration | Pearson correlation | .018 | .248^**^ | 1 | .095^**^ | .027^*^ | .029^*^ | .076^**^ | -.021 | -.057^**^ | -.069^**^ | -.037^**^ | .016 | .127^**^ |
|  | Sig. (2-tailed) | .122 | .000 |  | .000 | .017 | .011 | .000 | .068 | .000 | .000 | .001 | .156 | .000 |
|  | No. | 7678 | 7678 | 7678 | 7678 | 7678 | 7678 | 7678 | 7678 | 7678 | 7678 | 7678 | 7678 | 7678 |
| Maternal BMI | Pearson correlation | .162^**^ | .138^**^ | .095^**^ | 1 | -.032^**^ | -.005 | -.054^**^ | -.161^**^ | -.031^**^ | -.029^*^ | .016 | -.016 | .022 |
|  | Sig. (2-tailed) | .000 | .000 | .000 |  | .005 | .660 | .000 | .000 | .007 | .012 | .173 | .169 | .053 |
|  | No. | 7678 | 7678 | 7678 | 7678 | 7678 | 7678 | 7678 | 7678 | 7678 | 7678 | 7678 | 7678 | 7678 |
| Infertility factor | Pearson correlation | -.077^**^ | .041^**^ | .027^*^ | -.032^**^ | 1 | .036^**^ | .009 | .009 | -.020 | -.043^**^ | .048^**^ | .006 | .022 |
|  | Sig. (2-tailed) | .000 | .000 | .017 | .005 |  | .002 | .444 | .411 | .076 | .000 | .000 | .613 | .050 |
|  | No. | 7678 | 7678 | 7678 | 7678 | 7678 | 7678 | 7678 | 7678 | 7678 | 7678 | 7678 | 7678 | 7678 |
| EMT | Pearson correlation | .006 | -.059^**^ | .029^*^ | -.005 | .036^**^ | 1 | -.005 | -.009 | .005 | .002 | -.019 | .004 | .023^*^ |
|  | Sig. (2-tailed) | .630 | .000 | .011 | .660 | .002 |  | .670 | .451 | .647 | .876 | .099 | .744 | .042 |
|  | No. | 7678 | 7678 | 7678 | 7678 | 7678 | 7678 | 7678 | 7678 | 7678 | 7678 | 7678 | 7678 | 7678 |
| GN dose (IU) | Pearson correlation | -.180^**^ | .253^**^ | .076^**^ | -.054^**^ | .009 | -.005 | 1 | -.185^**^ | -.360^**^ | -.325^**^ | -.050^**^ | .130^**^ | .147^**^ |
|  | Sig. (2-tailed) | .000 | .000 | .000 | .000 | .444 | .670 |  | .000 | .000 | .000 | .000 | .000 | .000 |
|  | No. | 7678 | 7678 | 7678 | 7678 | 7678 | 7678 | 7678 | 7678 | 7678 | 7678 | 7678 | 7678 | 7678 |
| E_2_ on hCG day | Pearson correlation | .031^**^ | -.127^**^ | -.021 | -.161^**^ | .009 | -.009 | -.185^**^ | 1 | .523^**^ | .494^**^ | .060^**^ | -.083^**^ | .111^**^ |
|  | Sig. (2-tailed) | .006 | .000 | .068 | .000 | .411 | .451 | .000 |  | .000 | .000 | .000 | .000 | .000 |
|  | No. | 7678 | 7678 | 7678 | 7678 | 7678 | 7678 | 7678 | 7678 | 7678 | 7678 | 7678 | 7678 | 7678 |
| No. of oocyte retrieved | Pearson correlation | .156^**^ | -.232^**^ | -.057^**^ | -.031^**^ | -.020 | .005 | -.360^**^ | .523^**^ | 1 | .905^**^ | .017 | -.156^**^ | -.011 |
|  | Sig. (2-tailed) | .000 | .000 | .000 | .007 | .076 | .647 | .000 | .000 |  | .000 | .140 | .000 | .329 |
|  | No. | 7678 | 7678 | 7678 | 7678 | 7678 | 7678 | 7678 | 7678 | 7678 | 7678 | 7678 | 7678 | 7678 |
| No. of fertilized occytes | Pearson correlation | .148^**^ | -.207^**^ | -.069^**^ | -.029^*^ | -.043^**^ | .002 | -.325^**^ | .494^**^ | .905^**^ | 1 | .042^**^ | -.192^**^ | -.016 |
|  | Sig. (2-tailed) | .000 | .000 | .000 | .012 | .000 | .876 | .000 | .000 | .000 |  | .000 | .000 | .156 |
|  | No. | 7678 | 7678 | 7678 | 7678 | 7678 | 7678 | 7678 | 7678 | 7678 | 7678 | 7678 | 7678 | 7678 |
| Embryo type | Pearson correlation | .009 | .017 | -.037^**^ | .016 | .048^**^ | -.019 | -.050^**^ | .060^**^ | .017 | .042^**^ | 1 | -.024^*^ | -.279^**^ |
|  | Sig. (2-tailed) | .430 | .138 | .001 | .173 | .000 | .099 | .000 | .000 | .140 | .000 |  | .032 | .000 |
|  | No. | 7678 | 7678 | 7678 | 7678 | 7678 | 7678 | 7678 | 7678 | 7678 | 7678 | 7678 | 7678 | 7678 |
| Embryo quality | Pearson correlation | -.035^**^ | .032^**^ | .016 | -.016 | .006 | .004 | .130^**^ | -.083^**^ | -.156^**^ | -.192^**^ | -.024^*^ | 1 | -.030^**^ |
|  | Sig. (2-tailed) | .002 | .005 | .156 | .169 | .613 | .744 | .000 | .000 | .000 | .000 | .032 |  | .008 |
|  | No. | 7678 | 7678 | 7678 | 7678 | 7678 | 7678 | 7678 | 7678 | 7678 | 7678 | 7678 | 7678 | 7678 |
| No. of embryo transferred | Pearson correlation | -.055^**^ | .327^**^ | .127^**^ | .022 | .022 | .023^*^ | .147^**^ | .111^**^ | -.011 | -.016 | -.279^**^ | -.030^**^ | 1 |
|  | Sig. (2-tailed) | .000 | .000 | .000 | .053 | .050 | .042 | .000 | .000 | .329 | .156 | .000 | .008 |  |
|  | No. | 7678 | 7678 | 7678 | 7678 | 7678 | 7678 | 7678 | 7678 | 7678 | 7678 | 7678 | 7678 | 7678 |

Note: * *P* < 0.05; ** *P* < 0.01.

**SUPPLEMENTAL TABLE 2.**

Logistic regression analysis on the contribution of the potential predicting variables to implantation.

| **Variables** | **OR (95% CI)** | ***P*-value** | **Adjusted OR**  **(95% CI)^a^** | ***P*-value^a^** | **Adjusted OR**  **(95% CI)^b^** | ***P*-value^b^** |
| --- | --- | --- | --- | --- | --- | --- |
| **PCOS diagnosis** | 1.627 (1.368-1.935) | < 0.001 | 1.521 (1.274-1.816) | < 0.001 | 1.238 (1.030-1.489) | 0.023 |
| **Maternal age (y)** | 0.972 (0.959-0.984) | < 0.001 | 0.973 (0.960-0.985) | < 0.001 | 0.993 (0.978-1.008) | 0.355 |
| **Maternal BMI (kg/m^2^)** | 1.021 (1.004-1.038) | 0.013 | 1.020 (1.003-1.038) | 0.024 | 1.009 (0.991-1.027) | 0.340 |
| **Infertility duration (y)** | 0.986 (0.969-1.004) | 0.121 |  |  | 0.996 (0.977-1.015) | 0.680 |
| **Total dose of gonadotropin (IU)^1^** | 0.683 (0.646-0.723) | < 0.001 |  |  | 0.754 (0.708-0.803) | < 0.001 |
| **Serum E_2_ level (pg/mL) on hCG day^2^** | 1.046 (1.016-1.076) | 0.002 |  |  | 0.940 (0.908-0.972) | < 0.001 |
| **EMT (mm) on hCG day** | 1.087 (1.066-1.109) | < 0.001 |  |  | 1.093 (1.070-1.115) | < 0.001 |
| **No. of fertilized occytes** | 1.057 (1.047-1.067) | < 0.001 |  |  | 1.037 (1.025-1.049) | < 0.001 |
| **No. of embryos transferred** | 0.952 (0.888-1.022) | 0.173 |  |  | 1.114 (1.026-1.211) | 0.010 |
| **Embryo type** |  |  |  |  |  |  |
| Cleavage embryo | Ref | Ref |  |  | Ref | Ref |
| Blastocyst | 2.127 (1.754-2.580) | < 0.001 |  |  | 2.210 (1.797-2.719) | < 0.001 |
| **Embryo quality** |  |  |  |  |  |  |
| Cycle with high-quality embryos | Ref | Ref |  |  | Ref | Ref |
| Cycles without high-quality embryos | 0.211 (0.166-0.267) | < 0.001 |  |  | 0.270 (0.211-0.345) | < 0.001 |

Note: All the variables inputted in the model were shown in Supplemental Table 2. BMI=body mass index; CI=confidence interval; OR=odds ratio; E_2_=estradiol; hCG=human chorionic gonadotropin; EMT=endometrial thickness; Ref=reference; ^1^ per 1000 IU increased; ^2^ per 1000 pg/mL increased.

^a^Adjusted for maternal age and BMI.

^b^Adjusted for maternal age, BMI, infertility duration, total dose of gonadotropin, serum E_2_ level and endometrial thickness on hCG day, number of fertilized occytes, number of embryos transferred, embryo type and embryo quality.

**SUPPLEMENTAL TABLE 3.**

Univariable and multivariable regression analysis of risk of miscarriage, preterm delivery and pregnancy-induced hypertension stratified by lean vs. overweight/obese PCOS.

| **Outcomes** | **Classification** | **Variables** | **OR (95% CI)** | ***P*-value** | **Adjusted OR**  **(95% CI)^a^** | ***P*-value^a^** |
| --- | --- | --- | --- | --- | --- | --- |
| **Miscarriage** | **Lean** | **PCOS diagnosis** | 1.483 (1.094-2.012) | 0.011 | 1.599 (1.173-2.181) | 0.003 |
|  |  | **Maternal age (y)** | 1.077 (1.047-1.107) | < 0.001 | 1.077 (1.047-1.108) | < 0.001 |
|  |  | **Maternal BMI (kg/m^2^)** | 1.069 (1.021-1.119) | 0.004 | 1.042 (0.994-1.092) | 0.090 |
|  | **Overweight/obese** | **PCOS diagnosis** | 1.484 (0.861-2.558) | 0.155 | 1.467 (0.839-2.568) | 0.179 |
|  |  | **Maternal age (y)** | 1.017 (0.949-1.090) | 0.625 | 1.022 (0.952-1.097) | 0.548 |
|  |  | **Maternal BMI (kg/m^2^)** | 1.144 (0.986-1.329) | 0.077 | 1.129 (0.970-1.314) | 0.117 |
| **Preterm delivery (＜ 37 weeks EGA)** | **Lean** | **PCOS diagnosis** | 1.397 (1.067-1.829) | 0.015 | 1.360 (1.035-1.787) | 0.027 |
|  |  | **Maternal age (y)** | 0.984 (0.962-1.006) | 0.151 | 0.985 (0.962-1.008) | 0.194 |
|  |  | **Maternal BMI (kg/m^2^)** | 1.003 (0.966-1.042) | 0.870 | 1.004 (0.966-1.044) | 0.835 |
|  |  | **PIH** | 2.449 (1.542-3.890) | < 0.001 | 2.455 (1.544-3.901) | < 0.001 |
|  | **Overweight/obese** | **PCOS diagnosis** | 0.817 (0.465-1.434) | 0.481 | 0.753 (0.420-1.348) | 0.339 |
|  |  | **Maternal age (y)** | 0.988 (0.925-1.055) | 0.712 | 0.980 (0.916-1.049) | 0.564 |
|  |  | **Maternal BMI (kg/m^2^)** | 0.995 (0.853-1.159) | 0.944 | 0.993 (0.849-1.160) | 0.926 |
|  |  | **PIH** | 2.360 (0.854-6.520) | 0.098 | 2.550 (0.908-7.166) | 0.076 |
| **Pregnancy-induced hypertension** | **Lean** | **PCOS diagnosis** | 1.314 (0.626-2.759) | 0.471 | 1.403 (0.662-2.976) | 0.377 |
|  |  | **Maternal age (y)** | 1.038 (0.973-1.108) | 0.262 | 1.043 (0.976-1.115) | 0.216 |
|  |  | **Maternal BMI (kg/m^2^)** | 0.998 (0.896-1.112) | 0.974 | 0.983 (0.880-1.098) | 0.757 |
|  | **Overweight/obese** | **PCOS diagnosis** | 2.669 (0.964-7.390) | 0.059 | 2.765 (0.967-7.907) | 0.058 |
|  |  | **Maternal age (y)** | 1.044 (0.905-1.205) | 0.555 | 1.062 (0.917-1.231) | 0.419 |
|  |  | **Maternal BMI (kg/m^2^)** | 1.258 (0.949-1.668) | 0.111 | 1.220 (0.913-1.631) | 0.179 |

Note: All the variables inputted in the model were shown in Supplemental Table 3. BMI=body mass index; CI=confidence interval; OR=odds ratio.

^a^Adjusted for maternal age, BMI and PIH (only for preterm delivery).

**SUPPLEMENTAL TABLE 4.**

Logistic regression analysis on the contribution of QUICKI^1^ to pregnancy outcomes in PCOS women.

| **Pregnancy outcomes** | **OR (95% CI)** | ***P*-value** | **Adjusted OR (95% CI)^a^** | ***P*-value^a^** |
| --- | --- | --- | --- | --- |
| Clinical pregnancy | 1.058 (0.959-1.168) | 0.262 | 1.056 (0.956-1.166) | 0.287 |
| Miscarriage | 0.818 (0.705-0.950) | 0.008 | 0.816 (0.703-0.947) | 0.007 |
| Live birth | 1.145 (1.038-1.262) | 0.007 | 1.144 (1.038-1.262) | 0.007 |
| GDM | 0.854 (0.684-1.067) | 0.166 | 0.858 (0.686-1.072) | 0.178 |

Note: QUICKI=quantitative insulin sensitivity check index; GDM=Gestational diabetes mellitus.

^1^per 100 units increased; ^a^Adjusted for maternal age.
